# Burden of allergic disease among ethnic minority groups in high‐income countries

**DOI:** 10.1111/cea.14131

**Published:** 2022-04-14

**Authors:** Christina J. Jones, Priyamvada Paudyal, Robert M. West, Adel H. Mansur, Nicola Jay, Nick Makwana, Sarah Baker, Mamidipudi T. Krishna

**Affiliations:** ^1^ 3660 School of Psychology Faculty of Health & Medical Sciences University of Surrey Guildford UK; ^2^ Department of Primary Care & Public Health Brighton & Sussex Medical School Brighton UK; ^3^ 4468 Leeds Institute of Health Sciences University of Leeds Leeds UK; ^4^ Birmingham Regional Severe Asthma Service University Hospitals Birmingham NHS Foundation Trust Institute of Inflammation and Ageing University of Birmingham Birmingham UK; ^5^ Sheffield Children’s Hospital NHS Foundation Trust Sheffield UK; ^6^ Department of Child Health Sandwell and West Birmingham Hospitals Birmingham UK; ^7^ 531066 Anaphylaxis Campaign Farnborough UK; ^8^ Department of Allergy and Immunology University Hospitals Birmingham NHS Foundation Trust Institute of Immunology and Immunotherapy University of Birmingham Birmingham UK

## Abstract

The COVID‐19 pandemic raised acute awareness regarding inequities and inequalities and poor clinical outcomes amongst ethnic minority groups. Studies carried out in North America, the UK and Australia have shown a relatively high burden of asthma and allergies amongst ethnic minority groups. The precise reasons underpinning the high disease burden are not well understood, but it is likely that this involves complex gene–environment interaction, behavioural and cultural elements. Poor clinical outcomes have been related to multiple factors including access to health care, engagement with healthcare professionals and concordance with advice which are affected by deprivation, literacy, cultural norms and health beliefs. It is unclear at present if allergic conditions are intrinsically more severe amongst patients from ethnic minority groups. Most evidence shaping our understanding of disease pathogenesis and clinical management is biased towards data generated from white population resident in high‐income countries. In conjunction with standards of care, it is prudent that a multi‐pronged approach towards provision of composite, culturally tailored, supportive interventions targeting demographic variables at the individual level is needed, but this requires further research and validation. In this narrative review, we provide an overview of epidemiology, sensitization patterns, poor clinical outcomes and possible factors underpinning these observations and highlight priority areas for research.

**Key messages**

- Greater burden and poorer clinical outcomes among ethnic minority groups with allergic diseases are reported
- Reasons involve deprivation, literacy, proficiency of local language, access to specialists, cultural and religious beliefs
- Priorities include improved access/referral pathways, co‐produced and evaluated interventions, delineation of ethnicity‐specific phenotypes and endotypes

## 1 INTRODUCTION

Healthcare inequities and inequalities have been recognized as a major problem in high‐income countries (HICs) such as the UK and USA for over two decades. The COVID‐19 pandemic attracted major attention and renewed interest on this subject owing to disproportionate high mortality amongst patients from ethnic minority groups. Healthcare disparities have been linked to multiple socio‐demographic variables including age, gender, socio‐economic status, geographical location, cultural and religious factors.^1^ Evidence suggests a strong intersection between poor clinical outcomes and deprivation and literacy, as a significant proportion of the ‘most deprived population’ and those with poor general and health literacy are likely to be from ethnic minority groups.^2^ This is highly relevant in patients with allergies and allergic conditions as clinical outcomes depend on patient education and empowerment with implementation of self‐management plans. Whilst socio‐demographic variables are likely to strongly impact on clinical outcomes, it remains unclear at present if disease severity is intrinsically greater in ethnic minority patients resident in HICs.

This narrative review is structured to provide an overview of disparities in allergic diseases amongst patients from ethnic minority groups including epidemiological aspects, risk of sensitization and patterns and clinical outcomes. Reasons underpinning these disparities and mitigation strategies going forward to address gaps in research and healthcare are also incorporated in subsequent sections. The approach adopted by majority of studies presented in this review involves comparison of specific ethnic minority groups with reference white population.

## 2 ALLERGIC DISEASES IN ETHNIC MINORITY POPULATION IN HICS—DISPARITIES AND POOR CLINICAL OUTCOMES

Table 1 for an overview

**TABLE 1:** Summary of published ethnicity‐based disparities in allergic diseases in high‐income countries

	Key observations
Epidemiology	Higher incident risk^a^ of allergic rhinitis 2.36 (2.31–2.41), asthma 1.04 (1.01–1.07) and atopic eczema 1.66 (1.64–1.69) amongst British South Asians^3^ Higher incident risk^a^ of allergic rhinitis 2.56 (2.50–2.62) and atopic dermatitis 1.31 (1.28–1.33) amongst Afro‐Caribbean's and allergic rhinitis and 1.84 (1.80–1.89) and atopic dermatitis 1.33 (1.31–1.36) mixed ethnic groups in the UK^3^ Higher prevalence of asthma amongst Black population in USA (data as % (standard error)): White non‐Hispanic (NH; 7.7 (0.13)), Black NH 10.6 (0.36), Asian NH 3.8 (0.33) and Hispanic 6.6 (0.30) in the USA^86^ Higher prevalence of asthma in deprived population in the USA: most deprived, below 100% of poverty threshold 11.8 (0.63, std error) vs. 450% of poverty threshold 5.9 (0.26, std error)^86^ Delay and/or under‐recognition of allergic rhinitis amongst Black American children Higher risk of self‐reported food allergy amongst ethnic minority groups in the USA [Asian non‐Hispanic 1.28 (1.06–1.54, 95% C.I Black non‐Hispanic 1.20 (1.06–1.36, 95% C.I)]^18^ Higher risk of self‐reported food allergies amongst deprived population [1.08 (0.96–1.21, 95% C.I), 25,000–49,000 USD] in the USA^18^ Higher rates of single and multiple food allergies amongst Black American children vs. other ethnic minority groups (4.7% vs. 2.7%; *p* = .0001) considered as a single group)^70^ Higher standardized incident rates [58.3 (42.8, 76.3 95% C.I) per 100,000 person years] of community anaphylaxis amongst British South Asians compared with White population [31.5 (27.2, 36.3 95% C.I) per 100,000 person years)^31^ Higher rates of risk of food anaphylaxis amongst Asian children [adjusted OR 1.50 (1.16–1.94 95% C.I; *p* = .002)] compared with non‐Asian children in Australia^21^
Sensitization	Greater risk of aero‐allergen sensitization amongst African American children [2.17 (1.23–2.84, 95% C.I)] from deprived geographical locations^46^ Greater risk of cockroach sensitization amongst African American children [16.4 (4.8–55.9, 95% C.I)] and children [11.9 (4.30–40.80, 95% C.I)] from lower socio‐economic status^47^ Higher risk of sensitization to food allergens including shellfish, peanut, tree nuts, corn, legumes, milk, egg amongst Black American children
Clinical aspects	Higher rates of fatal asthma amongst Black NH patients vs. White non‐Hispanic patients (23.9 (0.76, std. error) vs. 9.9 (0.22, std. error) per million)^86^ Higher rates of fatal asthma amongst most deprived male British patients >45 years of age Higher rates of emergency room visits, hospital admissions and corticosteroid use amongst Black American patients Higher rates of emergency room visit for acute asthma amongst patient from most deprived areas in the USA Lesser proportion of British patients with severe asthma enrolled for biologic treatments Differences in proportion of Black American, Mexican American and Puerto Ricans children eligible for biologic therapies for severe asthma based on current selection criteria

^a^ Adjusted incident rate ratios (95% C.I).

### 2.1 Allergic rhinitis and asthma

Most published evidence on this subject comes from North America, with few studies from the UK, Europe and Australia. A recent large retrospective longitudinal cohort study in the UK primary care involving six million patients showed that the incident risk of common allergic diseases including allergic rhinitis, asthma and atopic eczema is greater amongst South Asians, Afro‐Caribbean's and those from mixed ethnic groups.^3^ In another longitudinal cohort study spanning over 25 years, the same authors showed that the long‐term risk of organ‐specific and systemic autoimmune disorders was significantly greater in the British patients with a pre‐existing allergic disease.^4^ These observations raise further questions regarding gene–environment interactions, as allergic diseases and autoimmune disorders are relatively less prevalent in low‐income countries and low‐middle‐income countries such as in the Indian subcontinent and Africa.^5^, ^6^

One study from the USA reported that the adverse impact of allergic rhinitis on African American children was greater than that reported amongst Latinos and non‐Latino white patients.^7^ There is similar evidence regarding a higher risk of atopy amongst South Asian British children.^8^ The Allergy and Infection study reported that Pakistani children were more likely to be sensitized to house dust mite than white children.^8^ Also, a greater proportion of children whose mothers were born outside the UK were sensitized to dust mite in comparison with those whose mothers were born in the UK.^8^

Data from the US Centers for Disease Control and Prevention (CDC) showed that the prevalence of asthma is greater amongst Black Americans, Puerto Ricans and mixed‐race patients in comparison with Hispanics, Asians and non‐Hispanic white patients over the last 2 decades.^9^ Furthermore, prevalence is greater in those belonging to lower socio‐economic strata. Asthma morbidity is also greater amongst Black American patients in comparison with white patients with respect to number of emergency room visits and hospitalizations. The US CDC 2017 data also reported higher rates of fatal asthma amongst Black non‐Hispanic patients in comparison with white non‐Hispanics, other non‐Hispanic and Hispanic patients (Table 1).^10^

Data from the UK severe asthma registry and the optimum patient care research database showed that, as compared to the white population, the ethnic minority population was more atopic, expressed higher type 2 inflammation markers and serum total immunoglobulin E (IgE), had lower lung function and worse asthma control.^11^ The refractory asthma stratification programme demonstrated that patients from ethnic minority groups were less likely to adhere to treatment advised in the clinical trial and had higher asthma exacerbations than white patients.^12^ Current guidelines for the use of biologics in asthma are based on data from translational research and clinical trials conducted mainly in white populations from HICs. Whilst ethnicity is considered to be mainly a social construct, a recent case‐control study from the USA involving African American, Mexican and Puerto Rican children highlighted key differences in blood parameters influencing eligibility for biologic therapies.^13^ Serum total IgE was significantly higher in Puerto Rican children compared with the other two groups. Peripheral blood eosinophil and neutrophil counts were significantly greater amongst Puerto Rican patients compared with African Americans. A greater proportion of Puerto Ricans were ineligible for anti‐IgE therapy in comparison with African American and Mexican patients. Similarly, a greater proportion of African American patients were deemed ineligible for eosinophil directed therapies compared with Puerto Ricans.^13^ This study highlights the need for (1) defining reference ranges for key blood parameters in ethnic minority groups, (2) developing selection criteria for biologic therapies to be more equitable and (3) ensuring drugs in development address the mechanism of disease in the non‐white population rather than assuming it is the same. Poor inclusion of the non‐white population in research results in the potential for white dominant therapies.

### 2.2 Food allergy and anaphylaxis

In the Learning Early About Peanut (LEAP) study, the relationship between skin prick test (SPT) response and specific IgE level to peanut differed significantly in the black population, the so‐called Simpson paradox (i.e. a statistical association or trend that appears for two groups with a particular variable is opposite when data for the two groups are combined). This may lead to over diagnosis of allergy if SPT profiles alone are used for black children. At study entry, participants were twice as likely to be in the peanut positive stratum if they were South Asian as white.^14^, ^15^ In the Enquiring About Tolerance (EAT) study, only 199 of the infants enrolled were non‐white compared with 1104 white participants. At enrolment, within group sensitization to one or more foods significantly differed by ethnicity—black/south Asian (48.6%), Mixed (22.9%) and white (12.3%).^16^ In the standard introduction group, the proportion of food allergy cases by ethnicity was 28.6% in the white study population and 71.4% in the non‐white study population, which was incongruent with the overall study population (84.7% white vs. 15.3% non‐white).^17^ A recent study from the USA noted higher rates of self‐reported food allergies amongst ethnic minority groups including Black and Asian patients, in comparison with white patients (11.2% and 11.4% vs. 10.1%).^18^ Furthermore, the authors reported that the prevalence of food allergies and other allergic conditions, such as asthma, eczema and allergic rhinitis, were significantly higher amongst deprived patients. Similar observations regarding greater prevalence and severity of food allergies, more frequent emergency room visits and food insecurity has been reported amongst ethnic minority groups in the USA and Australia.^16^, ^19^, ^20^, ^21^ A study from the USA, employing electronic medical records in 2.7 million patients, reported a higher prevalence of food allergies and intolerances amongst the Asian population.^22^ There is also some evidence regarding a higher rate of self‐reported peanut allergy, tree nut allergy and sea food allergy amongst Black American patients compared with white American patients.^18^, ^19^, ^23^, ^24^, ^25^ Interestingly, a rural–urban contrast with respect to food allergy prevalence was reported in a study from South Africa, with lower rates amongst children from rural communities.^26^

In the UK, Dias et al.^27^ established that 52.6% of paediatric allergy referrals were from a non‐white population compared with 35.9% in the general paediatric clinic and the complexity of allergic disease was greater in the non‐white population with them having more food allergens (2.05 vs. 1.22). Over a 14 year time period from 1990 to 2004, Fox et al.^28^ found that the proportion of children with peanut allergy from a non‐white heritage increased significantly from 26.8% to 50.31%, this was not the case in the white group or for egg allergy.

In a cross‐sectional survey in the USA involving 385 caregivers of Black and white American children with physician diagnosed food allergies, Vincent et al.^29^ reported association of knowledge, behaviour and attitude with socio‐economic status, ethnicity and clinical factors. Carers of Black children had comparatively lower knowledge scores regarding food allergy. Children of carers with higher food allergy knowledge scores were less likely to consume foods with precautionary allergy labels and more likely to consume allergen free foods. However, no differences were detected in emergency room visits between the two groups for severe food allergy reactions.^29^

The timing of food allergen introduction during infancy has an important implication on risk of food allergies during childhood. The food allergy outcomes related to white and African American racial differences cohort reported ethnicity‐based differences in the introduction of food allergens early in infancy amongst children with parent‐reported food allergies.^30^ African American children with an allergy to peanut, milk or egg were less likely to be introduced to respective allergens early in infancy compared with white children, although reasons underpinning the delay were not explored. Within the LEAP study, early introduction of peanut to high risk infants, between the ages of 4 and 11 months, was shown to significantly reduce the likelihood of peanut allergy at 60 months of age.^15^ When this primary outcome was stratified by race, peanut consumption was shown to be significantly more effective at reducing peanut allergy in the non‐white infants, whilst in the avoidance group, a higher percentage of non‐white infants developed peanut allergy. This raises questions as how best to target this intervention to ensure maximal benefit. The EAT study looked at early introduction of six allergens into an infant diet from 3 months of age to prevent food allergy. At the end of the study the intervention did not reach statistical significance for the primary outcome, but it was noted that the food allergy rates were higher in the non‐white participants, and that this group (especially South Asians), were less able to introduce new foods according to protocol in the weaning period.^17^ This again raises the question of how we can ensure appropriate education to these groups in any population‐based intervention.

Buka et al.^31^ systematically reviewed emergency room records of anaphylaxis occurring in Birmingham, UK and reported a higher rate in the British South Asian patients in comparison with white patients. The age‐standardized incidence rate for anaphylaxis and severe anaphylaxis was 58.3 (42.8–76.3, 95% C.I) and 20.4 (10.6–33.1, 95%, C.I) cases per 100,000 person years respectively for British South Asians as opposed to 31.5 (27.2–36.3, 95% C.I) and 10.7 (8.3–13.6, 95% C.I) per 100,000 person years respectively for white patients.^31^ Furthermore, this study also showed higher odds of severe anaphylaxis amongst patients <16 years old (2.37 [1.83–2.90)]. It also demonstrated that if you were from a non‐white population you were less likely to be referred to allergy clinic after an episode. Fatal anaphylaxis to food, medication and unspecified allergens in the USA were associated with African Americans and older age groups, and the incidence rate of fatal food anaphylaxis in African American males increased from 0.06 in 1999–2001 to 0.21 per million in 2008–2010.^32^ Asthma is an independent risk factor for anaphylaxis, and uncontrolled asthma in food allergy puts patients at an enhanced risk of severe anaphylaxis.^33^, ^34^ This is particularly relevant in ethnic minority groups as prevalence of asthma is significantly greater and a large proportion of cases are uncontrolled.

### 2.3 Atopic dermatitis

Studies have demonstrated that African American children have an increased risk of developing atopic dermatitis (AD) that the presentation varies dependent on skin colour, and that current scoring mechanisms will underestimate the severity of AD in black skin.^35^, ^36^, ^37^ The prevalence, persistence, severity and impact on health‐related quality of life of AD is greater amongst Black American patients and those from urban areas.^38^, ^39^, ^40^, ^41^ The US‐based studies have found that black children were less likely to see an outpatient provider for AD, but when they did they required more intensive treatment.^42^ In addition, non‐Hispanic black children and Hispanic children were more likely to have missed days of school, compared with non‐Hispanic white children, because of AD, which can affect learning and attainment and further impact on the cycle of deprivation.^43^ Filaggrin mutations are less frequent amongst Black patients. Also, there may be differences in *Staphylococcus aureus* colonization in skin between different ethnic groups. Some differences in phenotypes have also been described^44^, ^45^, ^46^; Black patients present with extensor dermatitis as opposed to flexural involvement, erythema may not be well delineated in black skin, and other differences have also been described, including peri‐follicular involvement, palmar hyper‐linearity, peri‐ocular dark circles and diffuse xerosis. It is important that these points are embedded into medical education to raise awareness amongst healthcare professional delivering allergy care.^44^, ^45^

## 3 FACTORS INFLUENCING OUTCOMES

**FIGURE 1:**
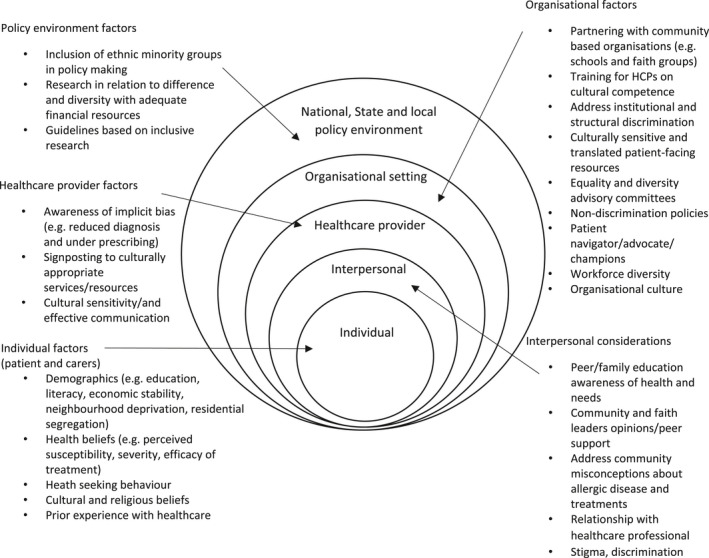
Factors to target to improve ethnicity‐based outcomes in allergic disease

Poorer clinical outcomes, such as those described above, are likely to result from a myriad of factors (Figure 1), with the literature documenting that access, treatment and outcomes vary by ethnicity. Canino et al state that asthma disparities have multiple, complex and interrelated sources including patient beliefs, health literacy and financial barriers to disease management.^47^ However, even before patient‐level factors have been considered, ethnic minority groups are often disadvantaged with regards to healthcare access and healthcare interactions, factors not unique to allergic disease. That said, socio‐demographic variables might influence the diagnosis and management of allergic airways diseases. A US study involving 275 children with no clinical and family history of allergic disease showed a two‐fold greater risk of sensitization to one or more aero‐allergens in African American children.^48^ Another reported that the risk of sensitization to cockroach was several‐fold greater (OR – 16.4 [4.8–55.9]) in African American children. The same study also showed a significantly higher risk of cockroach sensitization in children resident in urban locations (OR – 4.0 [4.0–10.7]) and that the risk was significantly greater in those living in deprived geographical locations (OR – 11.9 [4.3–40.8]).^49^ There is also evidence regarding under‐recognition and delayed diagnosis of allergic rhinitis in African American patients.^9^ This has important implications not only with respect to health‐related quality of life but also on the long‐term management of asthma, as allergic rhinitis is the most frequent co‐morbidity in asthma.

Similar observations have been reported by Asthma UK. There was a strong correlation between emergency room admissions and Index of Multiple Deprivation (IMD) average score as per the 2016–17 data set.^50^ The analysis of fatal asthma as per age and IMD quintile showed higher rates in men between 45–74 years and >75 years from the most deprived areas. Those from disadvantaged socio‐economic groups are more likely to be exposed to asthma environmental triggers such as allergens, cigarette smoke and air pollution. There is a significant variation in access to basic asthma care across geographical locations, age groups and ethnicity. The main causes of deaths identified in the National Review of Asthma Deaths were over reliance on short acting beta‐agonists and underuse of inhaled corticosteroids. This was driven by lack of provision of asthma self‐management plans, and poor patient knowledge and understanding of asthma and its associated risk. The Asthma UK report suggests suboptimal basic asthma care across the country.^50^ Furthermore, in the United States, after adjusting for other patient characteristics, black patients with asthma were less likely to see an outpatient physician than white patients,^51^ whilst others have shown healthcare utilization related to food allergy varies by ethnicity.^52^ There is also evidence that black and Puerto‐Rican children were absent from school more often because of asthma compared with white and Mexican children.^53^, ^54^ Moreover, the Yorkshire‐based Itchy, Sneezy and Wheezy project reported delays in referral of South Asian children to specialist allergy clinic compared with white children.^55^

Once within the healthcare system pathway, several further barriers become evident. Key barriers in UK‐specific studies include under‐diagnosis and reporting of asthma, lower prescription and lower use of beta‐agonists and inhaled corticosteroids, which may partly contribute to greater healthcare utilization.^56^, ^57^, ^58^ One factor which has been highlighted as contributing to under‐reporting and impact on prevalence of asthma is the misidentification of symptoms amongst South Asians and those from deprived backgrounds, leading to an underestimation of wheeze.^59^ Under‐reporting of asthma and wheeze may also be explained by poor or suboptimal local language proficiency of parents, with one study showing that participants requiring translation reported half the levels of asthma and wheeze compared with those parents who could respond in English.^60^ Factors at the individual‐level contributing to underuse of medication included beliefs that medication would cause more harm than good and a reluctance to disclose their child's asthma status.^61^ Furthermore, stigma of respiratory illness specifically related to erroneous beliefs about contagiousness of asthma were found to be present within Bangladeshi participants, but not amongst those who have migrated, thought to be as a result of acculturation.^62^

In a systematic review involving 25,755 children in 15 studies (2 in Pakistan, 5 in India and 8 in the UK), Lakhanpaul et al investigated facilitators and barriers in asthma care.^56^ The authors make a strong case to differentiate ethnicity‐based barriers to those that relate to minority ethnic group position *per se* at a population‐level (e.g. language‐related). As mirrored in the adult data, uncontrolled asthma in South Asian children has been linked to multiple ethnicity‐based barriers involving South Asian families, including denial regarding diagnosis, poor concordance, stigmatization regarding use of inhalers, self‐beliefs that asthma is a contagious disease and overreliance on emergency management in hospital rather than preventive therapy *via* primary care.

It is clear that ethnicity is confounded with social status, and that it can be difficult to determine the independent effects of each. Social and financial hardships, combined with management and environmental factors, explain much of the observed disparity in asthma‐related re‐admissions between black and white children in the United States.^63^ This is consistent with the notion that disparities arise from structural racism and social adversity. Those patients with more information, influence, resources and social networks may take more advantage of new technology and scientific development,^64^ which may further increase health disparities generally, a theory consistent with the Minority Stress Model.^65^ Non‐adherence to national asthma guidelines has been associated with patient's ethnicity^66^ which would have exacerbated ethnic disparities in a vicious cycle. There is evidence of lesser adherence to controller medication amongst ethnic minority groups with particular risk factors including race, education, income, baseline symptoms and attitude.^67^ Further evidence of lower adherence to prescription receipt, prescription initiation and medication use amongst ethnic minority groups has been reported, with medication beliefs and depressive symptoms acting as barriers, which may in part serve to reinforce the implicit bias of healthcare professionals to under‐prescribe beta‐agonists and inhaled corticosteroids if they believe their patients will not use them.^68^

## 4 WHAT CAN WE DO TO THESE REDUCE INEQUALITIES?

Some of the structural factors identified above (e.g. under‐diagnosis and low prescription rates) may reflect a lack of cultural competence which requires improved training and communication skills of healthcare professionals. The impact of institutional or systemic patterns of racism upon many allergic conditions is noted by Davis, and cultural competency training to combat these barriers and reduce implicit bias is advocated.^1^ That said, there is a paucity of high‐quality research showing a positive relationship between cultural competency training and improved patient outcomes which needs to be addressed by clearer descriptions and replicability of training curricula.^69^

The need for improved communication and education between health professionals and patients is evident from the factors stated above. In a qualitative study involving interviews with South Asian British mothers of children <5 years with food allergies, Peckover et al highlighted the need to raise awareness and improve knowledge amongst the South Asian community regarding allergies, as mothers sought help from family and friends as their first port of call regarding their child's health.^55^ Within anaphylaxis, several ‘Prevention of Future Death Reports’ highlight the fact that healthcare professionals should emphasize and ensure understanding for the requirement of adrenaline auto‐injectors to be carried at all times by patients and families.

The lack of diversity in research remains problematic with clinical trials failing to recruit a representative population, meaning new interventions may not be generalizable to certain ethnic minority groups. Again, this issue is not unique to allergic disease with relatively few clinical trials reporting complete ethnicity‐related data.^70^ That said, the challenges of recruiting to ethnic minority groups to asthma studies have been distilled to four key issues: patients where there are competing demands and inaccurate beliefs about diagnosis, institutions which have policies restricting incentives, research teams where staff training is needed, and interventions which may be unappealing or inconvenient to some.^71^ Also, the translation of patient‐facing materials carry significant cost implications; however, the cost of not doing this proactively can be seen throughout this paper Or article. One further barrier could be the need to balance scientific rigour with pragmatism as highlighted by Abrams, who suggests there is little focus on pragmatic research which permits variation in therapy to suit different ethnic groups.^72^ A way forward is to have proportionate representation of ethnic minority groups in clinical trials and translational research. However, sample size and heterogeneity would still not allow for meaningful sub‐analysis to be conducted. Standardization of international nomenclature with respect to ethnicity might create opportunities for systematic reviews and meta‐analysis. At present, national and international guidelines for allergic diseases are largely based on data generated from white population, thus not addressing disparities related to genetics and ethnicity.

## 5 LEARNING FROM RESEARCH FROM OTHER NON‐COMMUNICABLE DISEASES

As evidenced above, the importance of providing tailored health education (to both patients and healthcare professionals) is required on a number of levels (Figure 1). Existing theoretical models in health education have been criticized for emphasizing individual cognitive process without giving much attention to the embedment of cultural contexts and social structures in human behaviour.^73^ Indeed, a recent systematic review found few theory‐based asthma self‐management interventions for South Asians and African Americans.^74^ The importance of culture as a factor in health and health behaviours has been increasingly recognized, including its potential role in enhancing health messaging and interventions. The cultural characteristics of a group may have a direct or indirect relationship with health‐related priorities and decision‐making, as well as receptivity and adoption of health messages and interventions.^75^ It is therefore important that health programmes and interventions are culturally tailored not only to improve acceptance but also salience of health communication. Cultural tailoring of health information involves recognizing and reinforcing cultural values, beliefs, norms and practices of a group, and developing health messages based on these to provide context and meaning to the message.^76^

Community‐based culturally tailored interventions have been reported to be acceptable, feasible and effective in improving the management of various chronic conditions including cardiovascular disease and diabetes.^77^, ^78^ A recent systematic review suggests that interventions incorporating surface structure (tailoring intervention to observable characteristics of the target group) and deep structure (acknowledging the cultural, social, historical, environmental and psychological context of the target group that influences the health behaviour) are successful in improving disease awareness, healthcare access and self‐management.^75^, ^79^ The review highlights that, in addition to linguistically relevant materials, integration of deep structural components is key to intervention success. It suggests that awareness of fundamental philosophies of chronic disease self‐management, cultural practice of the target group, and involvement of families are important factors in intervention effectiveness. This was further emphasized in another review which recommends the provision of social support (deep structure) *via* linguistically and ethnically matched healthcare professionals, peers and family members as the most effective cultural component of the intervention in chronic disease.^80^ Identifying and measuring the cultural context of risks and resilience that influence disease is key to designing and evaluating culturally adapted interventions. Although surface variables (matching ethnicity and language) are important, cultural adaptations should focus on factors such as cultural norms, traditions and values that impact the intervention effectiveness.^81^

The use of culturally tailored interventions is increasing in community and healthcare settings, but clear and pragmatic guidelines for (co‐)developing, implementing and evaluating these interventions are lacking.^82^ In the current context of widening health disparities, strategic focus is required towards adopting culturally relevant programmes and practices to improve quality of care and promote health equity.

## 6 WHAT NEEDS TO CHANGE IN ALLERGIC DISEASE?

### 6.1 Service delivery aspects

To address the disparities above, several immediate and long‐term changes requiring a concerted effort from all stakeholders will need to be implemented. We need an ethnically diverse multi‐disciplinary workforce to build trust with communities and offer services that are accessible.^83^, ^84^ This is in keeping with recent results from the NHS staff survey. Inclusion of ethnicity‐based disparities in allergic disease within healthcare professional curricula and continuing professional development is imperative. This is to raise awareness not only of phenotypic differences in allergic disease, but also to identify, model and cultivate attitudes, behaviour and appropriate communication skills, that can help eliminate, rather than exacerbate, health and health care disparities.^85^ Inclusion of these disparities will enable a cultural transformation within health services to address systemic and structural racism and implicit bias. The need for healthcare professionals to acknowledge diversity within ethnic minority groups is required, with awareness that common cultural patterns may exist. Effective communication with diverse patient groups will be facilitated by the provision of accessible tools and resources, not least the availability of patient‐facing materials which are appropriate for different literacy levels, languages and cultures, given that these factors intersect and compound, leading to poorer clinical outcomes. Through consultation with patients and patient organizations and examination of ‘Prevention of Future Death Reports’, the need for materials which address perceptions of severity, vulnerability and stigma within the ethnic minority population are required, whilst also considering ways to empower patients (and their carers) to challenge healthcare professionals when it comes to appropriate prescriptions of emergency medication and referral to specialists.

### 6.2 Research initiatives

**FIGURE 2:**
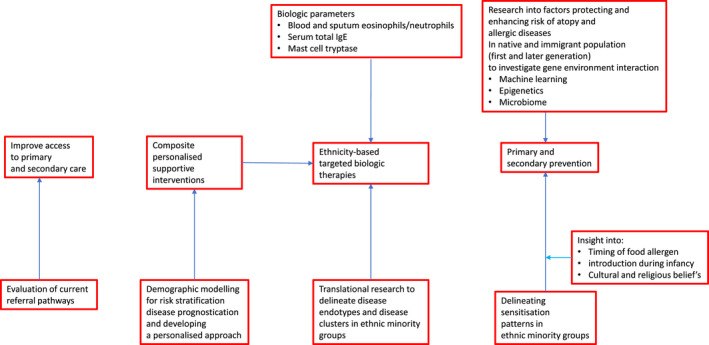
Integrated approach to improve clinical outcomes in allergic diseases in ethnic minority groups

Further research involving (a) UK‐based samples, (b) multi‐disciplinary expertise, (c) food‐allergy populations and (d) underrepresented ethnicities (e.g. Arabic and Chinese) is desperately needed, given that the majority of research included in this comprehensive review has been taken from asthma populations and North America studies. The latter being important given the differences in the US healthcare system (especially lack of access to care in respect to insurance coverage and ease of access to clinical facilities in underserved neighbourhoods) and lack of universal health care compared with the UK. There are a number of unanswered questions relating to epidemiology (e.g. what is the true prevalence of food allergy in ethnic minority groups within HIC and the UK specifically?) and phenotypes (e.g. is uncontrolled asthma in ethnic minority groups directly attributable to an individual's social context or is the disease intrinsically more severe?), in addition to those more behavioural in nature (e.g. how might cultural and religious practises impact on help‐seeking and referral pathways within ethnic minority groups as well as identifying the barriers and facilitators to effective self‐management?) (Table 2). The answers to these questions are likely to result in the development of composite supportive interventions which may be tailored to individuals based on their demographics and social context. It is likely that in time, artificial intelligence, in particular machine learning, will enable us to improve our ability to match interventions to particular subgroups. One hurdle which must be overcome in order to pave the way for these developments are a greater and proportionate representation of ethnic minority groups within clinical research. An integrated approach and strategy to improve clinical outcomes in allergic diseases is summarized in Figure 2.

**TABLE 2:** Evidence gaps and key research questions for addressing ethnicity‐based disparities in allergic diseases

Epidemiology	What is the prevalence of severe allergic rhinitis, asthma and atopic dermatitis amongst ethnic minority groups in high‐income countries? What is the incidence rate of anaphylaxis in ethnic minority groups in high‐income countries? What is the prevalence of true food allergy in ethnic minority groups in high‐income countries? Are there time trends in the incidence of allergic diseases by ethnicity? What is the effect of ethnicity, socio‐demographic status and rural/urban residency on the incidence and prevalence of allergic diseases?
Phenotypes	Are there differences in asthma phenotypes and disease clusters between white patients and patients from ethnic minority groups? Is uncontrolled asthma directly attributable to socio‐demographic variables (e.g. socio‐economic status, literacy, access to specialist, air pollution etc.) or is the disease intrinsically more severe in patients from ethnic minority groups? Are there differences in key blood parameters such as peripheral blood and sputum eosinophils/neutrophils and serum total IgE between white patients and those from ethnic minority groups?
Sensitization	Are there distinct sensitization patterns (considering both SPT and IgE) amongst ethnic minority patients in high‐income countries? What are the reasons underpinning higher risk of sensitization amongst Black American children? How are breastfeeding and weaning practices different in patients from ethnic minority groups and how might this impact on risk and patterns of sensitization to foods?
Care pathways	What are the facilitators and barriers in care pathways for allergic diseases with respect to patients from deprived geographical locations and those belonging to ethnic minority groups? Are there cultural, religious and health literacy barriers impacting on referral pathways amongst patient from ethnic minority groups?
Behavioural	How can we facilitate greater engagement within ethnic minority groups in clinical research? What are the barriers and facilitators to effective self‐management for those with allergic diseases from ethnic minority groups and the healthcare professionals who provide care? What behavioural/psychological constructs are important to address in ethnic minority groups to improve self‐management of allergic disease? What are the preferred mechanisms of health education delivery to encourage effective self‐management? How can we encourage healthcare professionals working with those with allergic disease from ethnic minority groups to be culturally competent?

## 7 CONCLUSION

It is clear from the published evidence that ethnicity‐based disparities in allergic disease exist and the problem is likely to be underestimated due to the factors and unanswered questions highlighted. Non‐white populations are an afterthought in research which has to change. Most research comes from outside of the UK and/or relates to asthma; proportionate representation of ethnic minority population in clinical trials and genome datasets, with translational research is desperately needed. There is encouraging research from other non‐communicable diseases that provision of tailored health education involving both surface structure (e.g. observable characteristics of ethnic minority) and deep structure (e.g. cultural, social, historical, environmental and psychological context of ethnic minority) are successful in improving disease awareness, healthcare access and self‐management. We therefore must involve community partners in designing disease prevention/ management plans, as well as in research more generally. Development, implementation and evaluation of cultural competency training is required for healthcare professionals, as are targeted biologic therapies for patients. A concerted effort from multi‐disciplinary experts will be required to address these disparities on a holistic scale.

## CONFLICT OF INTEREST

MTK is Chair of Equality, Diversity and Inclusion Working Group of BSACI. His department has received educational grants from ALK Abello, Allergy Therapeutics, MEDA and other pharma companies. MTK has received grants for work unrelated to this manuscript from NIHR, MRC CiC, FSA and GCRF. NM is Chair of the Paediatric Allergy Committee of BSACI. He has received educational grants (honoraria for educational lectures/attendance at allergy meetings) from ALK Abello, Nutricia, Abbott and Nestle. All other authors declare they have no relevant conflict of interest in relation to this publication.

## AUTHOR CONTRIBUTIONS

Jones and Krishna conceptualized the structured of the review. Jones, Krishna, Paudyal, West, Mansur, Jay and Makwana identified relevant literature for inclusion and contributed to original draft. All authors contributed to critical review of the manuscript, with relevant edits and expert opinion from specialist perspective. All authors approved the final manuscript as submitted.

## Data Availability

All data on which the manuscript is based on are presented within text and tables and appropriately referenced.
